# Risk assessment for the native anurans from an alien invasive species, American bullfrogs (*Lithobates catesbeianus*), in South Korea

**DOI:** 10.1038/s41598-022-17226-8

**Published:** 2022-07-30

**Authors:** Hye-Rin Park, Md Mizanur Rahman, Seung-Min Park, Jae-Hyeok Choi, Hee-Jin Kang, Ha-Cheol Sung

**Affiliations:** 1 Department of Biological Sciences·Biotechnology, Chonnam National University, 61186 Gwangju, South Korea; 2 Department of Biological Sciences, Chonnam National University, 61186 Gwangju, South Korea

**Keywords:** Conservation biology, Ecosystem ecology, Freshwater ecology, Invasive species, Wetlands ecology, Ecology, Zoology, Ecology, Environmental sciences

## Abstract

The invasive species are of global concern, and the Invasive American Bullfrog (IAB; *Lithobates catesbeianus*) is one of the worst invasive amphibian species worldwide. Like other countries, South Korea is also facing challenges from IAB. Although many studies indicated impacts of IAB on native anurans in Korea, the actual risk at the specific level is yet to evaluate. Considering the putative invasiveness of IAB, it is hypothesized that any species with the possibility of physical contact or habitat sharing with them, will have a potential risk. Thus, we estimated and observed their home range, preferred habitats, morphology, behavior, and ecology. Then, comparing with existing knowledge, we assessed risks to the native anurans. We found a home range of 3474.2 ± 5872.5 m^2^ and identified three types of habitats for IAB. The analyses showed at least 84% of native anurans (frogs and toads) were at moderate to extreme risks, which included all frogs but only 33% of toads. Finally, we recommended immediate actions to conserve the native anurans based on our results. As this study is the first initiative to assess the specific risk level from the invasiveness of *L. catesbeianus*, it will help the managers to set conservation priorities and strategies.

## Introduction

The organisms, which are settled in a region and environment, where they were introduced from outside, and posed threats to the native ecosystems, are known as alien invasive species^1–3^. The invasive animals cause multidirectional threats to the native species through predation, competition, hybridization, spreading the disease, etc.^4–7^. Recently, many studies highlighted immense negative impacts of invasive species on native biodiversity around the world^5,6,8^. In most cases, the introduction of alien invasive species is a result of human-mediated trespasses, especially in the form of pet and farm trades^9–12^. The Invasive American Bullfrog (IAB; *Lithobates catesbeianus*) is one of the perfect instances of such introductions that has spread over more than 40 countries across the continents and proved itself as one of the worst invasive amphibian species worldwide^13,14^.

Historically, IAB was exported as an ornamental and food item around the world^15^. Likewise, this species was imported to South Korea in the 1950s and 1970s primarily for food purposes from the USA and Japan^16–18^. The farming was not profitable due to a lack of taste and cost-effectiveness. To avoid further financial consequences, the IAB individuals were deliberately released into nature, which led to establishing local populations^19,20^. Furthermore, the IAB individuals were released regularly to nature as a part of religious and cultural beliefs until recently it was prohibited^21^. Although the first introduction of this species in South Korea was by Jinhae National Fish Farm, the first outdoor captive breeding was conducted in Chuncheon and subsequently established wild populations in the South-east of the country^17^. After being released, the physio-morphological characteristics, like big sized body, a huge number of eggs per clutch, food habits, etc., facilitate this species to invade new areas and outcompete the native species with negative impacts^22–24^. Together with other factors, e.g., climate change^25,26^, Korean fauna are experiencing immense stress from these kinds of invasive species. For instance, *Pelophylax nigromaculatus* and *P. chosenicus* are reported to be directly impacted by IAB^27,28^, while *Dryophytes suweonensis* indirectly^5^. Considering the raising conservation challenges for the native species from it, the South Korean Ministry of Environment has announced *L. catesbeianus* as an “ecosystem-disturbing species”^29^.

Although IAB is being considered a serious threat to the native amphibians in South Korea, the actual risk from it at the species level is yet to evaluate. Due to a lack of information on species-level risk from invasive species in places like South Korea, areas rich with invasive species threatened biodiversity do not match the hotspots of invasions^30^. Given consideration of invasive species as one of the primary causes of worldwide species extinctions but with lack of adequate information, assessment of species-level risk from the invasive species is very important^30,31^. Specifically, the amphibians, which hosted more than 50% of insular populations with invasive species threatened species, require a species-level risk assessment^32^.

However, the species-level risk for amphibians from IAB can be affected by the behavioral and microhabitat differences because primary threats may come from direct predation^33–35^. In addition, this invasive species is believed to eat whatever fits its mouth, i.e., larger IAB means ingestion of a larger amount and size of the prey species including native anurans^36–38^. Thus, we hypothesized all anurans, which share the habitat, breeding sites, or breeding seasons with this invasive species, are vulnerable, specifically from direct predation. To test this hypothesis, first, we estimated the home range of IAB and identified the habitat types in it. We also observed the food habits, movement patterns, breeding sites, and breeding seasons to compare them with previous literature to purify and enrich the dataset. Because, the home range, movement patterns, breeding behavior, and breeding seasons of IAB can be different based on geo-climatic conditions^39–42^. After the field observations, we matched this dataset with existing information for all anurans of South Korea to rank them according to the risk from IAB. As this study is the first initiative to assess the risk from the invasive species, *L. catesbeianus*, it will help the managers to set conservation priorities and strategies.

## Methods

### Study area

**Figure 1:**
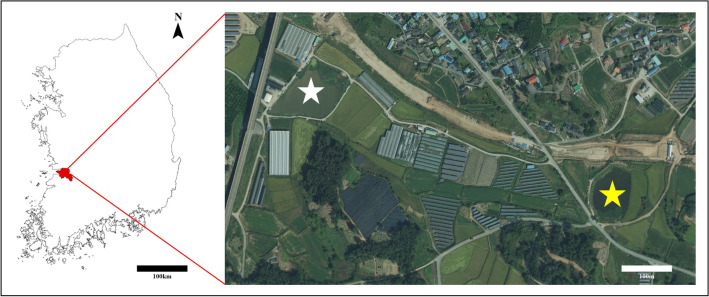
Map of the study area. The study sites are star marked, Deokro Reservoir (the white star), and Sango Reservoir (yellow star). The map was created using ArcMap (ver. 10.7, ESRI; https://support.esri.com/en/products/desktop/arcgis-desktop/arcmap/10-7-1) and QGIS Desktop TMS for Korean users Plugin (ver. 1.5; https://plugins.qgis.org/plugins/tmsforkorea/).

Depending on the pre-investigation concerning the existing IAB discovery points, we selected the research sites by considering the following characteristics; (i) Ensuring the sufficient presence of adult IAB, (ii) ease of geographical access to the study site, (iii) accessibility to the habitat around the reservoir, (iv) existence of adjacent reservoirs because of prior studies that IAB use several nearby ponds^40,43,44^. After a thorough search based on the above-mentioned criteria, we selected the Deokro (35°46′27″N 126°55′32″E) and Sango Reservoirs (35°46′20″N 126°55′56″E) locat ed in Hwangsan-dong, Gimje City, Jeollabuk province as our study sites (Fig. 1). The areas of Deokro and Sango Reservoirs are about 11,500 m^2^ and 6500 m^2^, respectively. The distance between the two reservoirs is about 640 m, where a two-lane paved road passed in between them. Both are artificial reservoirs surrounded by cultivated paddy and fallow lands. Among the amphibians, the Asiatic toad (*Bufo gargarizans*), Black-spotted pond frog (*Pelophylax nigromaculatus*), Japanese tree frog (*Dryophytes japonica*), Korean brown frog (*Rana coreana*), etc. were found to live in both reservoirs. In addition, the Gold-spotted pond frog (*P elo phylax chosenicus*), designated as Class II Endangered Wildlife was also found in and around the Deokro reservoir.

### Identification and selection of the individuals

All of the individuals were caught and released with permission from the Department of Environment, Gimje City, South Korea (Certificate No. Department of Environment-22817) and handled following the instructions and ethical permissions from the ethical committee, Laboratory Animal Research Center, Chonnam National University, South Korea (Certificate No. 2019-68). Furthermore, all other methods of this study were also performed in accordance with the relevant guidelines and regulations from the respective authorities. In addition, we followed the recommendations in the ARRIVE guidelines in our study^45^. After getting permission, we collected the IAB individuals from the wild. The sex of IAB was distinguished by mating call, the presence of a nuptial pad, and the size of the tympanum^46^. To catch male IAB, 10 traps (50 × 85 cm, fishing net) were installed where the mating call was heard. In the case of females, they did not give a mating call, so we caught them with our bare hands in reservoirs, surrounding waterways, and rice paddy. After catching the IAB individuals, we observed their morphological characteristics, like body weight (BW), body color, snout to vent length (SVL), head length, head width, size of the mouth, and tympanum and eye diameter, etc. The measurements of captured individuals were taken in units of 0.1 mm using vernier calipers (530 series, Mitutoyo, Japan), and BW was measured in units of 0.1 g using electronic scales (CUW-HX, CAS, Korea). Since this study was conducted on adult IAB, only individuals with an SVL greater than 75 mm were used for the study^43,44^.

### Radio-tracking

Radio-tracking was performed from June to October 2019. Following the previous studies, we divided a breeding season from June to August and a non-breeding season from September to October^43,47,48^. Considering the average lifespan of transmitters (BD-2, HOLOHIL, Canada) is 14 weeks, recapture was carried out after the last radio tracking in August after the three months of the first release. Additional secured or recaptured individuals were also released after the above procedure. We ensured that there was no abnormality in the movement of the individuals (sign of any traumatic condition) before release, and immediately after release. To avoid the influence on the movement of the individuals from the researcher's interference, we tracked them at least 3 days after release.

To distinguish the IAB individuals, a visible implant elastomer (VIE) tag (Northwest Marine Tech Inc, USA) injecting a fluorescent material was used^49^. The weight of the transmitter (BD-2, HOLOHIL, Canada) used for radio-tracking was 1.8 g. The maximum weight of the transmitter together with the belt, made to attach to the individual, was 5.4 g. This did not exceed 10% of the individuals’ weight, which is the recommended value for radio-tracking^50^.

**Figure 2:**
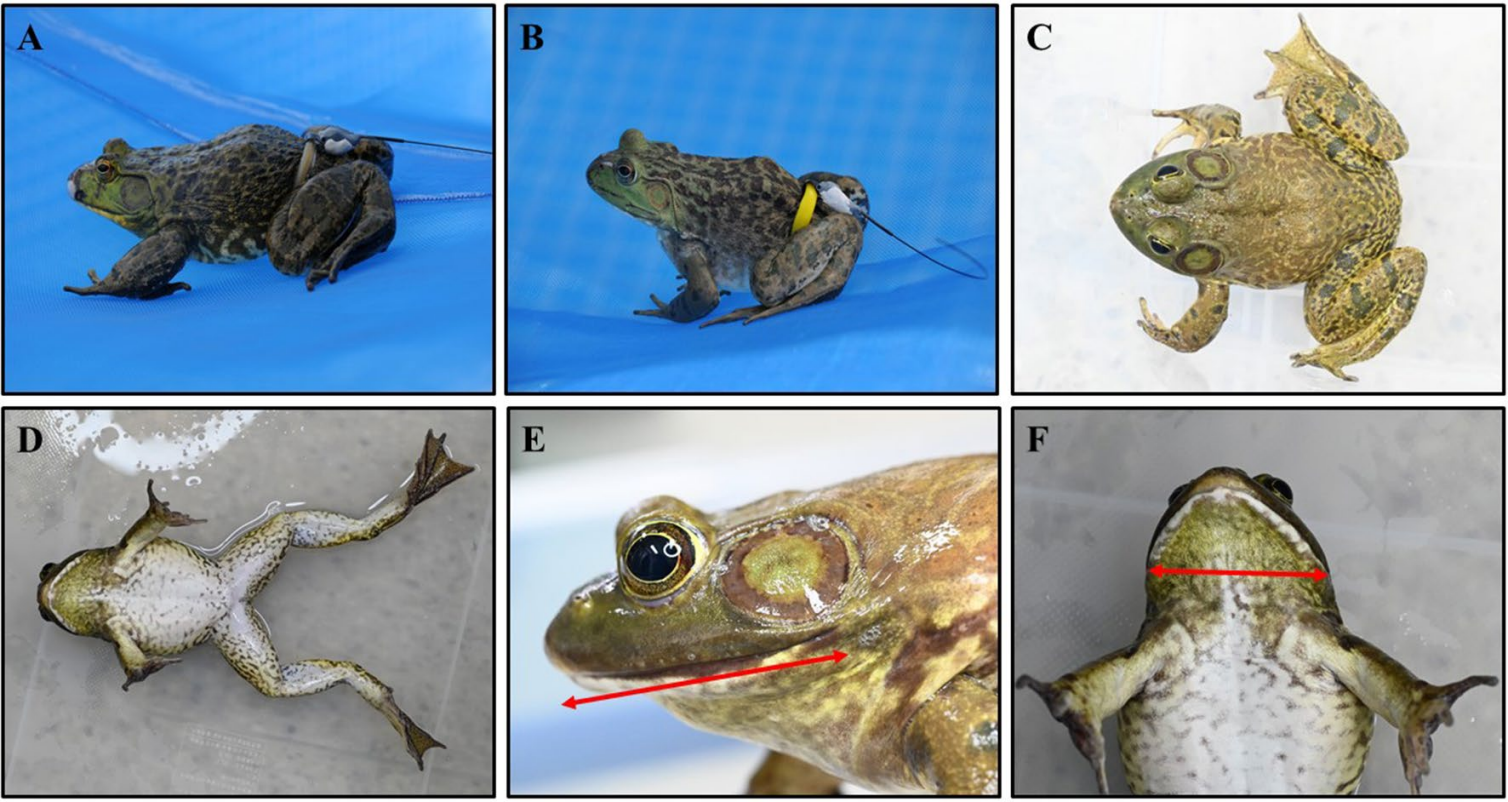
External features of *Lithobates catesbeianus* and the belt types used in radio-tracking- (**A**) dorsolateral view of a bullfrog with a latex belt, (**B**) dorsolateral view of a bullfrog with a stainless belt, (**C**) dorsal view, (**D**) ventral view, (**E**) mouth length, and (**F**) mouth width.

The transmitter fixing belt was manufactured to wrap around the waist of an individual^51,52^. To minimize irritation to the individual’s skin, a latex belt (LB; Standard Aoosung, Biogenetics, Korea) was used (Fig. 2A). The size of the latex ring is standardized (52 ± 2 mm). However, for some individuals that did not fit the size, a stainless steel string is wound around the specimen’s waist to make a custom belt (stainless belt). The stainless steel ring of the belt was completely wrapped with a rubber tube as we observed skin injuries in our preliminary experiments (Fig. 2B). After completing the above process, the IAB was observed for at least 5 days at Chonnam National University (Gwangju City) to check whether the belt was stably attached to the individual. For this purpose, a frame pool (450 × 220 × 84 cm, Intex, USA) was used, and during this period, males produced breeding calls, but no inclusion amplexus attempts or spawning were observed. After observations, we released the individuals into the study sites.

We determined the location of the individual using triangulation^53,54^. Since IABs mainly live in water^53,55^, when the individual could not be directly identified, we recorded their locations in the place with the strongest received signal strength^56^. We tracked them at 3-h intervals for 24 h starting at 15:00. Initially, we documented their points twice a week in the first month of release, June 2019. We found little movement in the IAB individuals, and hence, from July 2019, we performed tracking once a week.

We selected the method of displaying the points on a map in the field by confirming that the errors of the GPS device were often irregular through a preliminary survey. To minimize the subjective difference of the researcher who records, a grid at 5 m intervals was created and used on the map using ArcMap (ver. 10.7, ESRI). If no signal is obtained for more than 2 weeks, in consideration of the possibility of long-distance spread of IABs, we conducted radio-tracking at night in all reservoirs within a 1.6 km radius^57^, known as the maximum distance traveled by IABs, from the study reservoir.

### Estimation of home range and statistical analyses

We used ArcMap and QGIS Desktop (ver. 2.18, QGIS Development Team) to calculate the distance traveled by the IAB. We calculated the distance between two consecutive points with an interval of 3 h and did not include the distance between the last point of the 24-h tracking and the next 24-h tracking in the analysis. We analyzed the IAB's home range as MCP (minimum convex polygon) using ArcMap's HRT (home range tool)^58^. The obtained data (all individuals, between sexes, and breeding and non-breeding seasons) did not show normality, so we used the Mann–Whitney U test for all analyses using IBM SPSS statistics (ver. 20, IBM).

### Risk assessment

We have checked our observation data with previous studies and included some of the information like food items, breeding seasons, etc. of IAB. Furthermore, we mined the previous literature to get relevant information on all of the South Korean anurans. Primarily, we have followed Lee et al. for the species information^48^. In addition, we reviewed other articles, including Werner et al., Yoo et al., Chung, Hirai, Quagliata et al., Lee and Park, Wang and Li, Kim et al., Jancowski and Orchard, An and Waldman, Borzée et al., Park et al., Borzée et al., Park et al.^24,59–70^. Initially, we searched for all of the information that might influence competition, predation, and disease contamination by the IAB. Depending on the availability, we gathered species information on ten different criteria (Appendix 1). Finally, due to the lack of enough information for all of the species, we selected four criteria to assess the risks from *L. catesbeianus*.

We assessed the risks estimated from the criteria: (a) SVL, (b) Habitats, (c) breeding sites, and (d) breeding seasons. We characterized these categories into subgroups as follows-SVL: we considered the 40 mm mouth size a threshold as we did not find a smaller mouth size than that in subadult and mature individuals and thus the subgroups were (i) ≤ 40 mm, (ii) 41 – 50 mm, (iii) 51– 60 mm, and (iv) ≥ 61 mm; Habitats: we found three types of habitats within the home ranges of IAB, (i) reservoir (permanent water bodies), (ii) marsh and waterways (shallow waters and the water channels), and (iii) paddy fields and fallow lands (paddy plantation areas and unused lands). Thus, categorized the native anurans into four groups, the species that use (i) all types of habitats, (ii) two of the three habitat types, (iii) one of the habitat types, and (iv) different habitat types than the IAB’s habitats; Breeding sites: depending on our observations and literature reviews, we categorized the breeding sites into- (i) rivers and reservoir, (ii) water pools, marsh and agricultural waterways, and (iii) paddy fields and fallow lands, and graded following the procedure of habitat use; Breeding seasons: the breeding season of IAB lasts for five months (April–August), 150 days. Thus, we categorized the species into-(i) completely fall within these months, (ii) at least 100 days fall within this period, (iii) less than 50 days fall within this period, and (iv) did not fall within these months. We scored all categories with 25 points. Each of the categories was scored with four grades, A, B, C, and D (Table 1). Thus, the total score was 100, and the species were ranked into four categories according to their scores. We considered the species at Extreme Risk (ER) if it obtained scores of 76–100, High Risk (HR) for 51–75, Moderate Risk (MR) for 26–50, and Lower Risk (LR) for 0–25 (Table 1).

**Table 1:** The criteria and process of grading and scoring for risk assessment for the anurans from the American bullfrogs in South Korea. Here, SVL = Snout to vent length; A, B, C, D are grades and the numbers in the parentheses are the representative scores.

Categories/Grades	A (25)	B (16.67)	C (8.33)	D (0)	Standardized criteria in bullfrogs
SVL	≤ 40 mm	41–50 mm	51–60 mm	≥ 61 mm	Considering the mouth size is more than 40 mm
Habitats	All habitats	Two habitats	One habitat	Completely different	(i) rivers and reservoir, (ii) water pools, agricultural waterways, and marshes, and (iii) paddy fields and fallow lands
Breeding site	All habitats	Two habitats	One habitat	Completely different	(i) rivers and reservoirs, (ii) water pools and agricultural waterways, and (iii) rice paddies on flat land
Breeding season	Completely within the period	51–100 days	≤ 50 days	Completely different	April–August

## Results

### Morphology

We captured a total of 18 individuals (8 males and 10 females). The mean SVL of all subjects was 128.2 ± 11.5 mm (n = 18, range: 104.7–145.0 mm), and the mean BW was 222.2 ± 52.6 g (n = 18, range: 136.6–314.7 g). The head was almost one-third of the body’s length. The tympanum was big, almost twice the eye diameter, and conspicuous in males. The males also can be distinguished from the females by having nuptial pads on their hands and yellow color throats. Supra-tympanic membrane and the longitudinal folds were present. The mouth was very big, starting from beyond the midpoint of the tympanum and extending to the pointed snout (Fig. 2E,F). It was ranging from around 40–60 mm. A Black rounded pupil was surrounded by a golden circular iris. Fully webbed hind limbs (190 mm) were larger and stronger than the web-free forelimbs (80 mm). The hind limb stretched beyond the snout. The dorsal color varied from pale to dark green, with dark and pale brownish to yellowish bands. The ventral side is white to yellowish white with green and brown spots. The ventral side of the throat was almost white in the females, while green or yellow in the males. The lateral sides were yellow (Fig. 2C,D).

### Home range

**Figure 3:**
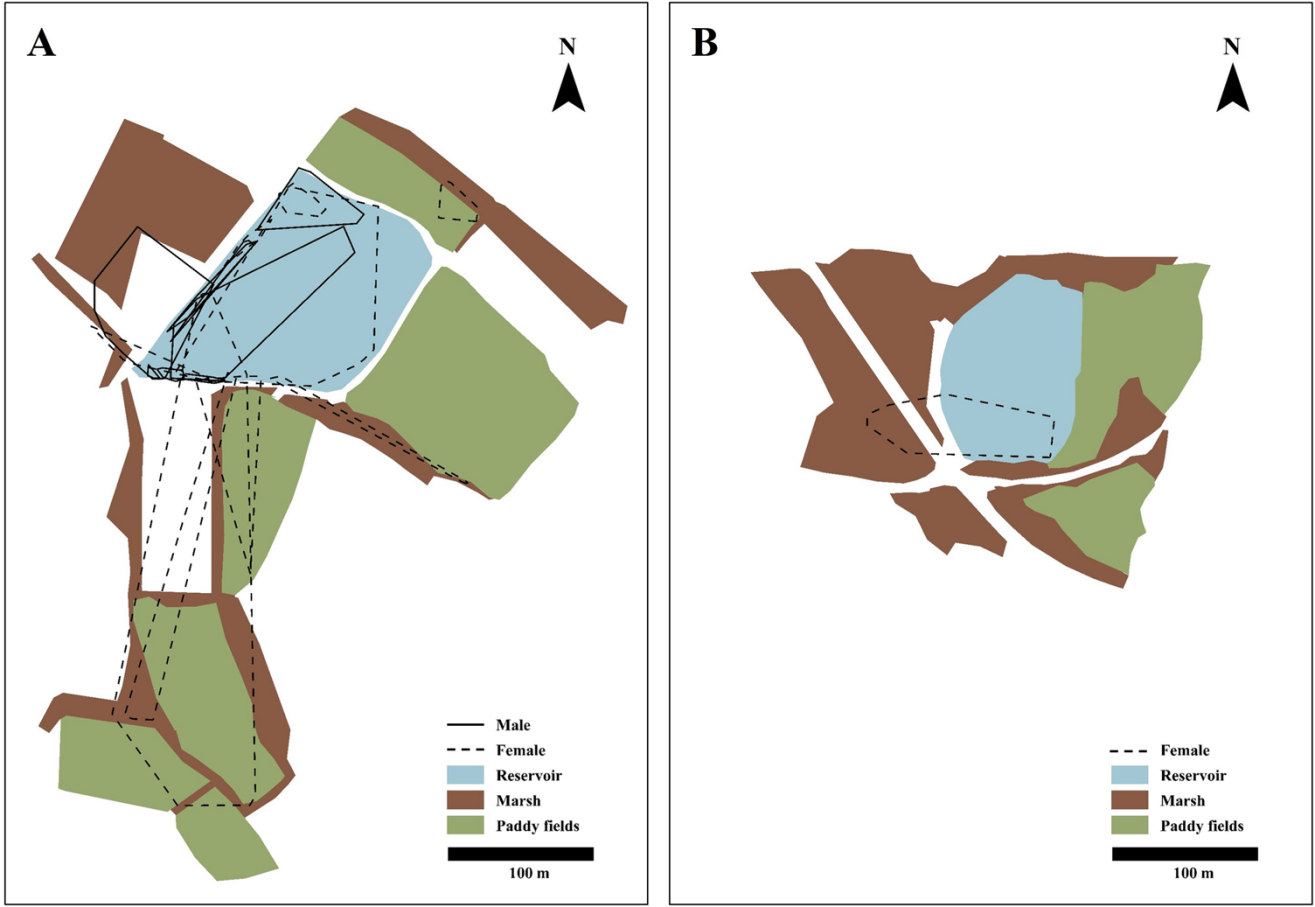
Home range of *Lithobates catesbeianus* in (**A**) Deokro and (**B**) Sango Reservoir in Hwangsan-dong, Gimje City, Jeollabuk Province, South Korea.

The mean home range of the IAB (MCP 95%) was 3474.2 ± 5872.5 m^2^ (n = 18, range: 36.3–23,089.8 m^2^; Fig. 3; Table 2). The mean area of the males was 1699.0 ± 2392.7 m^2^ (n = 8, range: 36.3–5752.1 m^2^) and 2358.9 ± 7459.6 m^2^ (n = 10, range: 179.1–23,089.8 m^2^) for females. There was no statistically significant difference in the home ranges between sexes (*df* = 17, Z = − 1.510, *p* = 0.131). For the home ranges in different seasons, the breeding season had a mean home range of 2339.0 ± 5423.9 m^2^ (n = 15, range: 36.3–21,592.1 m^2^), and non-breeding season had a mean home range of 1855.4 ± 2308.0 m^2^ (n = 8, range 25.4–5186.0). We did find any significant difference in the home ranges between seasons (*df* = 22, Z = − 0.258, *p* = 0.796).

**Table 2:** Home range of *Lithobates catesbeianus* in Gimje, South Korea.

Group	n	SVL (mm) mean (± SE)	BW (g) mean (± SE)	MCP 95 (m^2^) mean (± SE)
Male	8	119.5 (3.5)	197.1 (18.6)	1699.1 (845.9)
Female	10	135.2 (2.3)	242.2 (14.4)	4894.2 (2358.9)
Total	18	128.2 (2.7)	222.2 (12.4)	3474.1 (1384.2)

In breeding season, mean home range of the males was 555.8 ± 657.5 m^2^ (n = 7, range: 36.3–1708.0 m^2^) and 3899.4 ± 7246.1 m^2^ (n = 8, range: 338.5–21,592.1 m^2^) for females. There was a significant difference between sexes (*df* = 14, Z = − 1.967, *p* = 0.049) during the breeding season. In non-breeding season, the mean area of males was 3294.9 ± 2843.1 m^2^ (n = 3, range: 25.4–5186.0 m^2^) and 991.6 ± 1671.5 m^2^ (n = 5, range: 47.1–3969.9 m^2^) for females. During this period, there was no significant differences in home ranges between them (*df* = 7, Z = − 0.745, *p* = 0.456; Table 3).

**Table 3:** Comparison of home ranges and movement patterns of *L. catesbeianus* between sexes and periods.

Season	Statistical parameters	Male – Female	Breeding season	Non-breeding season
**Home ranges**				
Breeding season	*df*	14	–	22
	Z	− 1.967	–	− 0.258
	*p*	0.049*	–	0.796
Non-breeding season	*df*	7	22	–
	Z	− 0.745	− 0.258	–
	*p*	0.456	0.796	–
Breeding – Non-breeding Season	*df*	17	–	–
	Z	− 1.510	–	–
	*p*	0.131	–	–
**Movement patterns**				
Breeding season	*df*	557	–	927
	Z	− 1.450	–	− 3.561
	*p*	0.147	–	0.000*
Non-breeding season	*df*	369	927	–
	Z	− 0.525	− 3.561	–
	*p*	0.600	0.000*	–
Breeding – Non-breeding Season	*df*	927	–	–
	Z	− 0.429	–	–
	*p*	0.668	–	–

‘*’ = The difference is significant at the 0.05 level (2-tailed).

### Movement patterns

The movement distance of the IAB varied from 0 to 133.9 m. The mean movement distance was 3.2 ± 9.0 m (n = 928). The movement distances in males and females were 2.3 ± 5.0 m (n = 389) and 3.8 ± 11.0 m (n = 539) respectively. The sexes showed no statistically significant difference in the movement patterns (*df* = 927, Z = − 0.429, *p* = 0.668). Whereas, the mean movement distances between breeding (3.4 ± 9.1 m; n = 558) and non-breeding (2.8 ± 9.0 m; n = 370) seasons significantly differed (*df* = 927, Z = − 3.561, *p* = 0.000). However, there was no significant difference in the movement distance between males and females during the breeding period (*df* = 557, Z = − 1.450, *p* = 0.147) as well as during the non-breeding period (*df* = 369, Z = − 0.525, *p* = 0.600; Table 3).

### Habitat use

**Figure 4:**
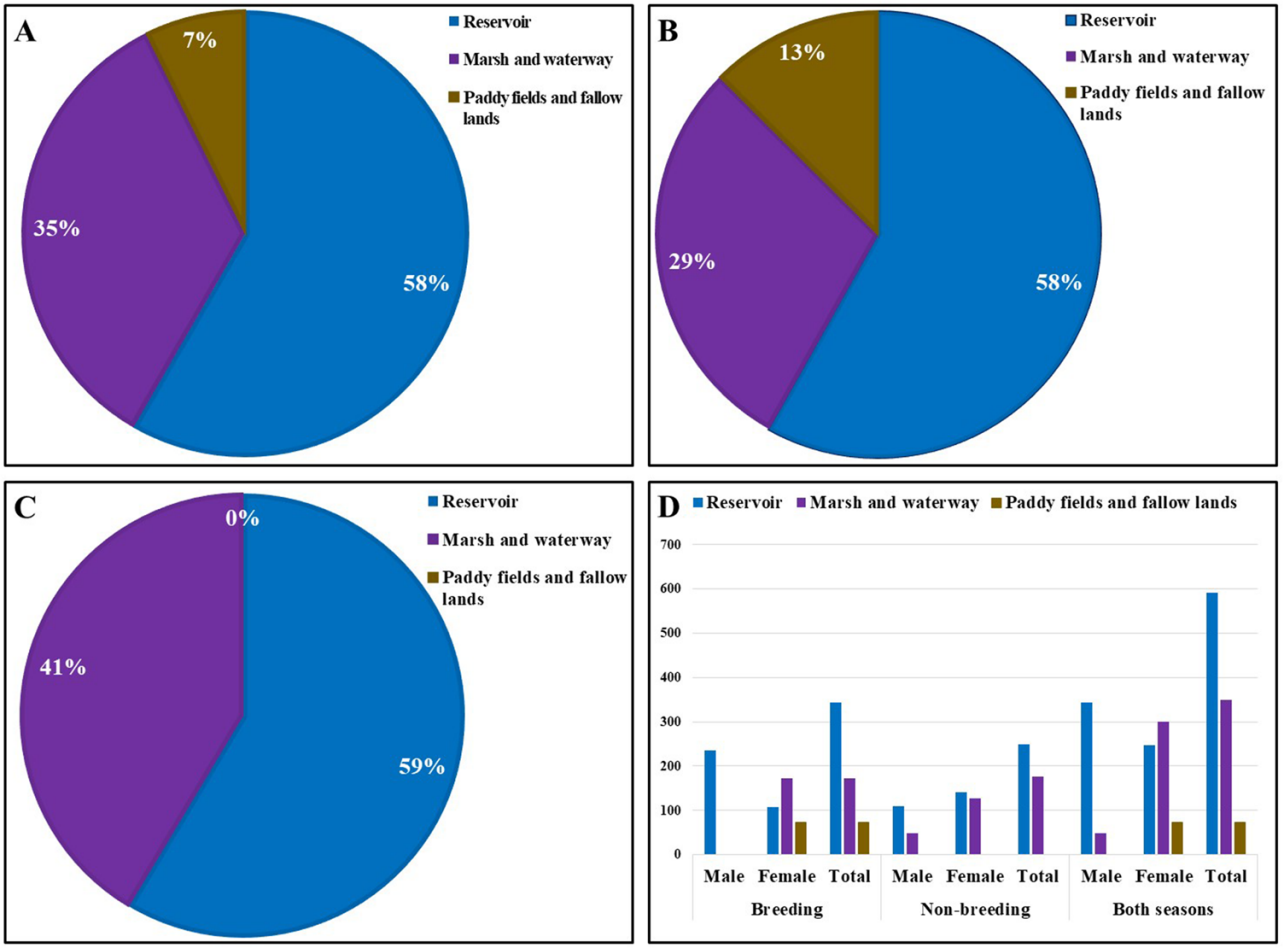
The proportion of habitat use by *Lithobates catesbeianus* within their home range in the -(**A**) breeding and non-breeding seasons, (**B**) breeding season, (**C**) non-breeding season, and (**D**) comparison of habitat use by male–female individuals during breeding and non-breeding seasons.

The analyses showed that the reservoirs are the most preferred habitat type for the IAB. There were 58% points recorded by the radio telemetry from the reservoir during this study. The marsh and the waterways were the second preferred habitat type (35%), while paddy fields and fallow lands were the least preferred having as little as 7% points in the radio-tracking (Fig. 4A). In terms of sexes, females used diversified habitats than males. During the study periods, the percentages of using habitats by females were 40%, 48%, and 12% for reservoirs, marsh, and paddy fields respectively (Fig. 4D). Whereas, males did not use the paddy fields and passed almost all the time in the water reservoir (88%; Fig. 4D).

However, the proportion of using reservoirs in the breeding and non-breeding seasons were almost similar (58% and 59% respectively; Fig. 4B,C), while in the breeding season, IABs (both sexes) did not use the paddy fields and fallow lands (Fig. 4C). In both breeding and non-breeding seasons, females spent less time in the reservoirs (30% and 52% respectively) than males (100% and 69% respectively). In contrast to using the reservoirs, females used marsh habitat in both breeding and non-breeding seasons (49% and 48% respectively) more than males (0% and 31% respectively). The paddy fields and fallow lands were unused during the non-breeding seasons, while only females used this habitat type in the breeding season for a small portion (21%; Fig. 4D).

### Threat assessment for the native anurans

**Figure 5:**
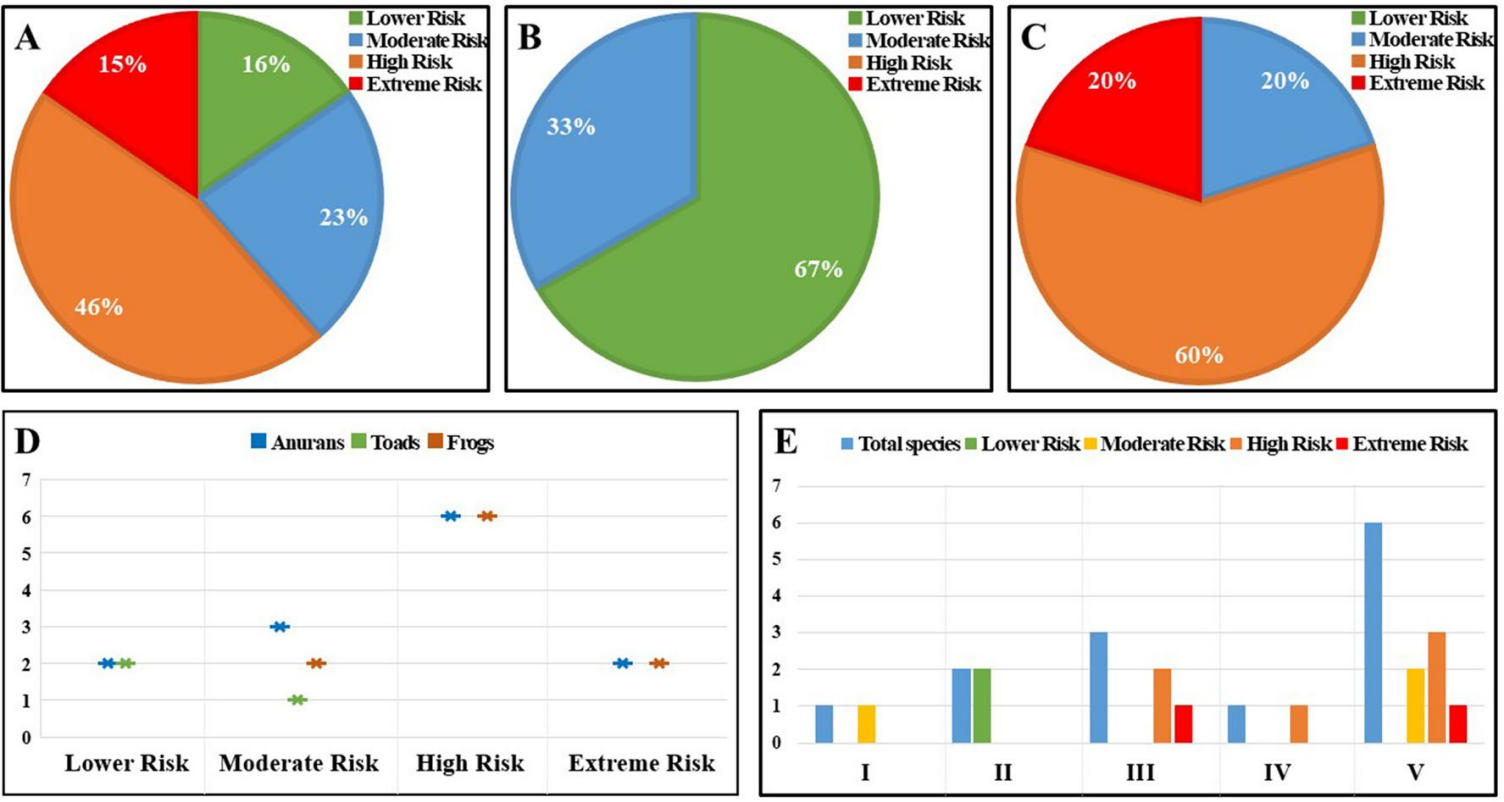
Risk assessment of the anurans of South Korea from the invasion of American bullfrogs-(**A**) all anurans, (**B**) toads, (**C**) frogs, (**D**) comparison of anurans by risk categories, and (**E**) comparison of risk statuses of anurans by family. Here, I = Bombinatoridae, II = Bufonidae, III = Hylidae, IV = Microhylidae, and V = Ranidae.

We assessed risks from the IAB for 13 anurans, three toads, and ten frogs, in South Korea (Table 4). The analyses showed that 84% of anurans are at risk from this invasive species. Only 16% of species were at LR, while 46% were at HR followed by MR and ER (23% and 15% respectively; Fig. 5A). Among the toads, 67% were at LR and 33% were at MR (Fig. 5B). For the frogs, we found only 20% of the frog species at MR. Despite having higher scores, most of the species were measured as HR ranked (60%; Table 4). In addition, there were 20% of species evaluated as ER (Fig. 5C). Overall, frogs were more vulnerable than the toads to the invasiveness of the IAB in South Korea (Fig. 5D). In terms of family, Ranidae had the maximum number of species, most of which were evaluated as highly vulnerable due to the presence of the IAB. Among the six species, three were evaluated at HR, two at MR, and one at ER. Hylidae had two species with HR and one estimated at ER. The only species of Microhylidae was evaluated as of HR. Both of the species from the toad family Bufonidae were measured as LR, while the only species of Bombinatoridae was considered as MR (Fig. 5E).

**Table 4:** Risk statuses of the anurans of South Korea from the invasion of *L. catesbeianus*. ‘*’ marked species were found in the study areas coinhabiting the bullfrogs.

Family	Scientific Name	Common Name	Risk Score	Risk Status	IUCN status	Remarks
Bombinatoridae	*Bombina orientalis*	Oriental Fire-bellied Toad	41.67	MR	LC	–
Bufonidae	Bufo gargarizans*	Asiatic Toad	16.67	LR	LC	–
	*Bufo stejnegeri*	Water Toad	0	LR	LC	–
Hylidae	Dryophytes japonica*	Japanese Tree Frog	100	ER	LC	–
	*Dryophytes suweonensis*	Suweon Tree Frog	75	HR	EN	Class I Endangered Wildlife
	*Dryophytes flaviventris*	Yellow-bellied Tree Frog	66.66	HR	–	–
Microhylidae	*Kaloula borealis*	Boreal Digging Frog	75	HR	LC	Class II Endangered Wildlife
Ranidae	*Glandirana rugosa*	Wrinkled Frog	75	HR	LC	–
	Pelophylax chosenicus*	Gold-spotted Pond Frog	91.67	ER	VU	Class II Endangered Wildlife
	Pelophylax nigromaculatus*	Black-spotted Pond Frog	66.67	HR	NT	–
	Rana coreana*	Korean Brown Frog	58.34	HR	LC	–
	*Rana huanrenensis*	Huanren Brown Frog	33.33	MR	LC	–
	*Rana uenoi*	Korea Large Brown Frog	41.66	MR	–	–

## Discussion

We assessed specific-level risk for the anurans from IAB in South Korea. For the risk assessment, we have studied the home range of IAB in Korea. Home ranges could be varied with the geographic localities and environments^42,71^. Thus, it is important to study the home range of invasive species in newly invaded areas to know their basic ecology and possible impacted environments and ecosystems there. The IAB is one of the highly discussed amphibian invasive species, especially for their potential threats to the native species, in Korea. Yet, we know very little about their home range, habitat sharing, and actual risk to the species level.

In the present study, we found a mean home range of 3474.2 ± 5872.5 m^2^ for the IAB in South Korea (Table 2). The result varied from the home range studies in different parts of the world^42,44^. For instance, it was larger than the studies from California, USA (1600 m^2^)^44^, while smaller than the individuals in Grote Nete, Belgium (11,086.73 m^2^)^42^. Similar trends were found in the movement patterns. We detected a maximum daily movement in our study at 196.6 m, while Descamps and De Vocht found 742.21 m^42^. These variations could be attributed to the duration of study periods and methods of data collection. Cooper (2017) studied only three months, which is a quite smaller period to estimate the home range^44^, while Descamps and De Vocht used a time interval of about 12 h to take the data^42^. In our study, we collected the weekly data for five months at regular 3-h intervals. Thus, this study was more precise and accurate than the previous literature. Furthermore, we found no significant difference between the movement patterns of the male and females in breeding and non-breeding seasons. These observations are similar to Descamps and De Vocht (2016), based on radio-telemetry, and different from Louette et al. (2013), based on capture-recapture methods, indicating the importance of using radio-telemetry in studying home range movement patterns^41,42^. However, the movements among the IABs were significantly higher in the breeding season than in the non-breeding season (Table 3). It could be attributed to the migration pattern, environmental conditions, habitat types, and/or finding mating partners. Previous studies also found similar results and assumed a link between this kind of migration pattern and the environmental conditions and the habitat types^39,42,71^.

In our study areas, we found three types of habitats, reservoir, marsh, and paddy fields, used by the IAB. Although Descamps and De Vocht found two types of habitats for IAB in their study areas, the nature of habitats was the same^42^. The first two habitat types of our study were similar to their observation, while the third kind, paddy fields, and fallow lands, was nearby the reservoir with a wet environment. Apart from nature, the use of habitat types may depend on the availability of food sources and the presence of water. In addition, sexes and seasons could also be a determinant^40,41,72^. Although a few studies suggested adult males travel to seasonal pools^40^, we found females to explore more habitat types than males. Especially, during the breeding seasons, males tended to remain in the reservoir, the breeding place for the IAB (100%; Fig. 4D; Appendix 2). This result was supported by many other literature^42,72^. This might be attributed to the lack of parental care and post-natal duties for females, to have a greater chance to get the right mate choice for avoiding inbreeding, to enhance the breeding success, and to the territorial behavior of the male IAB^72^. Due to having a spectacular feeding behavior and eating capability, beyond the purposes of migration, the presence of IAB in a habitat type makes the native species vulnerable^15,73^.

The native species that share the habitat could be vulnerable to the invasiveness of IAB through competition, predation, and disease transmission^73–76^. In the present study, we could not include the risk of disease transmission due to the lack of enough information on disease susceptibility for all anurans in South Korea. Our results suggested most of the anurans (61%; Fig. 5A) are at high to extreme risk from the invasion of IAB. This finding is in accordance with other alien invasive species risk assessments for the vertebrates worldwide^30,32^. Accordingly, authors suggested invasive species as one of the major causes of the amphibian declines and extinctions^31^. Similar to the present study, previous studies found more than 50% of insular and 23% of endangered (IUCN category) amphibian species to be threatened by the invasive species^30,32^. Although we evaluated a fewer number of traits and scored all equally, the method was capable to assess species-level risks from the IAB locally. Scoring all scores equally may lead to a possible bias, for instance, the SVL. We scored an SVL of less than 40 mm, irrespective of other traits, vulnerable as it may fit into the IAB’s mouth. It may not be vulnerable if it does not come in contact with the IAB. Considering the aquatic habitat dependency of IAB and other native anurans, at least during breeding seasons, we ignored the possibility of these biases in our analyses.

However, the analyses showed frogs are more vulnerable to the negative impacts of IAB than toads. This is attributed to the differences in habitat preferences and the presence of poison glands (e.g., parotid glands). This finding is consistent with the previous studies^34,35^. During the field works, we observed the IAB was engulfing an Oriental fire-bellied toad *(Bombina orientalis*) individual. After a few moments of engulfing, the toad was spewed out, which we assumed might be because of the poisons. However, Park et al. found most of the amphibians eaten by the IAB in Gyeongsangnam province, South Korea, were Asiatic toads (*Bufo gargarizans*), indicating high adaptability and rapid change in food tastes of the IAB^24^. The food tastes might be dependent on the availability of the food sources. The larval stages also could be an important food source, which is yet to evaluate. Thus, we considered toads being less vulnerable due to direct predation from the IAB cautiously pending further study.

Unlike toads, frogs do not have poison glands. Additionally, sharing the habitat types and miniature sizes make frogs more vulnerable to the invasiveness of the IAB^73^. Previous studies also suggested similar results^5,73^. In accordance with Doubledee et al., we also found a ranid species, *Pelophylax chosenicus*, at ER from the presence of IAB^73^. This species is already of high conservation concern being categorized under ‘Vulnerable’ (VU) and ‘Class II Endangered Wildlife’ according to the Korean Ministry of Environment^77,78^. The vulnerability of this species might be attributed to similar habitat choices as the IAB^35^. Other ranids were also ranked as moderate to high risk, possibly due to their size and probability of physical confrontation with the IAB. Accordingly, the only microhylid, *Kaloula borealis*, which is a ‘Class II Endangered Wildlife’ in South Korea, ranked as HR. Although tree frogs use mostly different habitat types than ranids and microhylids, the analyses showed the hylids to be under extreme risk from the invasiveness of *L. catesbeianus*. This could be attributed to their miniature sizes and using the same breeding sites. Previous studies also found similar conservation risks for this gro up of frogs^5^.

Given the knowledge gap in the species-specific risk status for the presence of IAB in South Korea, the present study may serve as a guideline. This study intensifies the concept of the negative impact on the native species from the alien invasive species. This study suggests the invasive species may impact adversely all native species indiscriminately (84% of anurans are at moderate to extreme risk). However, as ER category of this study showed, special attention is needed for those species which are already endangered due to other conservation issues. Depending on the assessment of Korean anuran vulnerability to the invasiveness of the IAB, we call for immediate action to control and eradicate *L. catesbeinus* populations from nature. We recommend species-specific management and conservation plans according to their risk status.

## Supplementary Information

Supplementary Information 1.

Supplementary Information 2.

## Data Availability

All data generated or analyzed during this study are included in this published article [and its supplementary information files].
