# Supplementary material for: Risk assessment for the native anurans from an alien invasive species, American bullfrogs (Lithobates catesbeianus), in South Korea
Source: Sci Rep. 2022 Jul 30;12:13143. doi: 10.1038/s41598-022-17226-8 (PMC9338931; doi:10.1038/s41598-022-17226-8)
Supplement: Supplementary file 1 — Supplementary Information 1. [file 41598_2022_17226_MOESM1_ESM.docx]

**Appendix legends**

**Appendix 1.** The species information that used for assessing the threats from the invasion of *Lithobates catesbeianus*.

**Appendix 2.** Radio-tracking data for estimating the home range of the American bullfrogs in Gimje, South Korea.
